# A Comparative Exploration of Quality Assurance Results by the Third-Party Pharmaceutical Education Evaluation in Japan

**DOI:** 10.3390/pharmacy9010006

**Published:** 2020-12-31

**Authors:** Kayoko Takeda, Naoko Arakawa

**Affiliations:** 1 Department of Pharmaceutical Education, Faculty of Pharmaceutical Sciences, Hokkaido University of Science, Sapporo 006-8585, Japan; 2 School of Pharmacy, University of Nottingham, Nottingham NG7 2RD, UK; Naoko.Arakawa@nottingham.ac.uk

**Keywords:** quality assurance, Japanese six-year initial pharmacy education, Third-Party Pharmaceutical Education Evaluation

## Abstract

Background: The Standards for the Establishment of Universities in Japan were revised; subsequently, the number of schools or universities of pharmacy/pharmaceutical sciences increased from 46 in 2002 to 74 in 2016.The pharmacy education programme was also changed from four to six years, which was implemented in 2006. In this study, we provide the comparative results of the first cycle of the third-party accrediting organization, the Japan Accreditation Board for Pharmaceutical Education (JABPE); Methods: The results of the first cycle of all universities or schools of pharmacy assessed by the JABPE from JABPE website were retrieved, and we collated and compared the results based on the 13 areas of the assessment standards; Results: In “improvements”, the number of public universities or schools was less than that of private universities or schools, and the number of old private universities or schools was also less than the number of new private universities or schools in all assessment areas. Conclusions: These results suggest that new universities or schools established since 2003 have not yet established their own quality assurance mechanism within the institutions. We need to review the Japanese pharmacy education system or the assessment criteria for it to bring about essential change.

## 1. Introduction

Professional education is required to be based on societal needs and/or national policy in order to effectively serve local and national population, and any professional area involves a complex and dynamic interplay among practice, regulation and education [1,2]. As the regulatory bodies for professional education are responsible for serving and protecting patients and the public interest, the regulatory bodies are accountable for assuring the professional education preparing graduates meeting societal needs and expectations. At the same time, it is likely that developments, innovations, or initiatives in one area will lead or drive change in others. For example, new regulation may be required to bring about change required in practice, whilst beneficial innovations in practice may result in changes in regulations. The International Pharmaceutical Federation [1] states that education has been a strong driver of change in practice, but education may sometimes lag and be disconnected from proactive engagement with practice, and education and regulation can be drivers of quality improvement in education. In addition, it is important to ensure that none of the “gaps” described above become too wide at any point [1].

In 1991 and the 2000s, the standards for the Establishment of Universities in Japan were revised, detailed statements about academic programmes were abolished, and the fundamental criteria were relaxed [3,4]. Subsequently, the number of schools or universities of pharmacy/pharmaceutical sciences increased from 46 in 2002 to 74 in 2016 [5,6]. There are currently 74 pharmacy/pharmaceutical universities or schools, and approximately 11,500 students enter to pharmacy/pharmaceutical schools per year in Japan [7]. The Japanese School Education Act was also revised in 2004, and pharmacy education was changed from a four-year pharmacy education programme to a six-year programme, which was implemented in 2006 [8,9]. A model core curriculum for six-year pharmacy education programme was developed to guide the change in the educational system by Ministry of Education, Culture, Sports, Science and Technology (MEXT) [8]. This substantial reform indicated that becoming a pharmacist requires not only sufficient practical training but also needs to demonstrate contribution to patient care as a professional [9]. Currently, initial Japanese pharmaceutical education consists of two programmes: a six-year programme intended to initially train pharmacists and a four-year programme primarily intended to train pharmaceutical scientists. However, after this model core curriculum was implemented, several problems were identified (e.g., there was insufficient time to incorporate the characteristics of each university due to a tight schedule, the competencies needed as a pharmacist were not clearly stipulated, etc.), resulting in a revision that involved an approximate 25% reduction in the amount of content, which was then implemented in 2015 [9]. The main purpose of the revised model core curriculum was to foster 10 elements of “Professional Competencies for Pharmacists”, which were stipulated as follows: (1) professionalism; (2) patient-oriented attitude; (3) communication skills; (4) interprofessional collaboration; (5) basic sciences; (6) medication therapy management; (7) community health and medical care; (8) research competency; (9) lifelong learning; and (10) education and training [9]. All students are required to obtain these competencies by graduation. To support students’ development of the competencies, the six-year programme in Schools of Pharmacy/Pharmaceutical Sciences deliver the model core curriculum, covering (A) Philosophical Principles for the Education of Student Pharmacists, (B) Pharmaceutical Sciences in Society, (C) Fundamentals of Pharmaceutical Sciences, (D) Health and Environmental Sciences, (E) Therapeutics: Clinical Pharmacology, Pharmacotherapy, and Pharmacokinetics, (F) Pharmacy Practice Experiences, and (G) Research [8,9]. Pharmacy students are awarded with a Bachelor of Pharmacy (B. Pharm.) degree following the six-year programme.

When the initial pharmacy education moved to the six-year system, Government enforced the new law regarding the quality assurance for pharmacy education. All six-year pharmacy programmes require to be accredited by the third-party accrediting organization, the Japan Accreditation Board for Pharmaceutical Education (JABPE). The JABPE was established in 2008 [10,11]. The JABPE, which is the first of its kind to specifically evaluate and accredit health professional education in Japan, stated their evaluation process in 2012, and has completed the first cycle of the evaluation of all six-year initial pharmacy education programmes in March 2020. The process of the evaluation and accreditation of the pharmacy programmes was called the “Third-Party Pharmaceutical Education Evaluation” in Japan [10]. All School of Pharmacy/Pharmaceutical Sciences must undergo evaluation every 7 years, and the results of these evaluations are granted at three levels, including “full accreditation”, “probational accreditation”, and “non-accreditation”, which are publicly informed. In addition, the JABPE’s Third-Party Pharmaceutical Education Evaluation provides assessment feedback at three levels, including: “merits”, “recommendations” and “improvements” to universities and schools. “Merits” are given when items meet the standards, “recommendations” are items that meet the minimum standard for passing and require more effort until the next cycle. “Improvements” must improve within 3 years of the year following the assessment, and evidence-based documents must be submitted to the JABPE. Schools of Pharmacy/Pharmaceutical Sciences that are indicated as “non-accreditation” or “probational accreditation” must improve these areas within 3 years of the year following the assessment and submit evidence-based documents to the JABPE. However, even if universities have obtained “full accreditation”, if there are problems, “improvements” or “recommendations” are identified by feedback of the JABPE, and must also submit the above documents to the JABPE. The submitted documents are reviewed by the council and published on the website of the JABPE. The purposes of the evaluation are as follows: (1) to ensure the quality of education programmes, (2) to promote their improvement, and (3) to provide support for actively obtaining public understanding and cooperation. The evaluations cover performance of students and structure of each education programme, which divided into 13 areas that comprise 57 standards and 176 indicators [10,11]. The number of schools keep increasing in Japan as the Standards for the establishment of universities relaxed, and It has already been announced that three new universities and schools of pharmacy will be established after 2021 as of November 2020. Currently, the total population in Japan is declining, and the elderly population is increasing. According to the population estimate of the Statistics Bureau of the Ministry of Internal Affairs and Communications (confirmed in February 2020), the total population of Japan is 126 million, and the number of people aged 65 and over is 36 million, an increase of 311,000 compared to the same month of the previous year [12]. This means that the number of university entrance candidates will show a greater decline in the near future. The trend of massification of higher education resulted in many issues across the world, including the lowering prior academic achievement of the student at the admission [13,14]. In order to ensure and maintain the quality and obtained competencies of pharmacy graduates while increasing access to the pharmacy initial education, it is important to ensure and improve the quality of the education. In particular, the number of private pharmacy university/school has increased 1.5-fold in Japan, due to the relaxation of the standards for the establishment of universities, while the number of public pharmacy/Pharmaceutical university/school has not changed. In terms of the pass rate of the national pharmacist examination, the number of examinees enrolled before 2002 was about 10,000, 90% of whom passed, but now it is about 70% for more than 14,000 examinees. This means, although the number of examinees has increased, the number of people who have passed the national examination has hardly changed. In other words, these issues might be caused by the relaxation of the standards for the establishment of universities [15]. In this study, we provided the comparative results of the first cycle of the JABPE’s Third-Party Pharmaceutical Education Evaluation, in which all 74 pharmacy universities or schools were assessed and identify quality issues among Japanese universities or schools of pharmacy.

## 2. Materials and Methods

All 74 universities or pharmacy/pharmaceutical schools were assessed by the JABPE’s the Third-Party Pharmaceutical Education Evaluation from 2015 to March 2020. The process of JABPE’s evaluation is conducted by visits of 6 experts, together with evidence submitted by the assessed school. We retrieved the results of the first cycle of all universities or schools of pharmacy assessed by the JABPE from JABPE website [10], and we collated and compared the results based on the 13 areas of the assessment standards (Table 1). The assessment of the 13 areas included the following: 1. Mission and Goals, 2. Organization for Curriculum, 3. Basic Contents of Medical Profession Education, 4. Pharmaceutical Education Curriculum, 5. Pharmacy Clerkship, 6. Education for Nurturing Problem Solving Ability, 7. Admission Policy and System for Acceptance, 8. Grading/Promotion/Graduation, 9. Student Services, 10. Teacher Organization/Staff Organization, 11. Institutions/Facilities, 12. Collaborative Relationships with Society and 13. Self-check/Self-evaluation. We collated which and how many universities received the comments in “improvements” and “recommendations” for each assessment area in all 74 universities or schools. Collected data were analysed in order to explore the differences between public and private universities, or old private (established before 2003—when the Standards for the establishment of universities relaxed in Japan) and new private universities (established after 2003) for investigating any patterns of accreditation results based on the university/school establishment or ownership. This is based on the general consideration in Japan that deviation score of public schools is better than private schools, and our hypothesis that the relaxation of the Standards for the establishment of universities would have led to the difference in quality. For these categorical data (public and private or old private or new private), a chi-square test for trends was used for analysis using SPSS Statistics 25.0 (IBM, Chicago, IL, USA), and the significance level was set to *p* < 0.05.

## 3. Results

### 3.1. Assessments of All 74 Universities and Colleges

In the first cycle, 6 of the 74 universities or colleges were “probational accreditation”, and 70 universities or schools were rated as “full accreditation”, and six universities or colleges were re-evaluated by JABPE (not every 7 years) (data not shown). The JABPE identified “improvements” items in more than 60% of universities or schools in the following 7 of 13 areas: “2. Organization of Curriculum” (45 universities or schools, 60.8%), “3. Basic Contents of Medical Profession Education” (70 universities or schools, 94.6%), “4. Pharmaceutical Education Curriculum” (57 universities or schools, 77.0%), “5. Pharmacy Clerkship” (62 universities or schools, 83.8%), “6. Education for Nurturing Problem-Solving Ability” (71 universities or schools, 95.9%), “8. Grading/Promotion/Graduation” (70 universities or schools, 94.6%), and “13. Self-check/Self-evaluation” (65 universities or schools, 87.8%) (Table 1). The JABPE also identified “recommendations item” in more than 60% of universities or colleges, including all items except “11. Institutions/Facilities” and “12. Collaborative Relationships with Society” (Table 1). For “recommendations item”, most areas were identified by the JABPE (as “improvements”) for universities or schools of pharmacy except for “11. Institutions/Facilities” (Table 1 and Supplementary Table S1).

### 3.2. Comparing the Assessments of 17 Public Universities or Schools with the Assessments of 57 Private Universities or Schools

To know presently the state of Japanese pharmacy education, we compared public universities and private universities. For “improvements”, the significant differences between the 17 all-public and 57 all-private universities or schools were “2. Organization of Curriculum”, “7. Admission Policy and System for Acceptance” and “10. Teacher Organization/Staff Organization”, with chi-square test *p* values of 0.001, 0.001, and 0.01, respectively. There is no item referred as “improvements” nor “recommendations item” for “12. Collaborative Relationship with Society”, both in public and private universities or colleges (Table 2).

With regard to “4. Pharmaceutical Education Curriculum” and “9. Student Services”, the number of public universities or colleges that were identified by the JABPE (as “improvements”) was slightly higher than the number of private universities or schools (Table 2), while the number of areas of public universities or schools that were identified by the JABPE was less than those of private universities or schools (Table 2).

### 3.3. Comparing the Assessment of 29 Old Private Universities (Established before 2003) or Colleges with 28 New Private Universities or Colleges (Since 2003)

To know the differences in private schools considering the effect of relaxation of the Standards for the establishment of universities, we compared public universities and private universities. For “improvements”, the significant differences between 29 old private and 28 new private universities or schools were “4. Pharmaceutical Education Curriculum”, “7. Admission Policy and System for Acceptance”, and “10. Teacher Organization/Staff Organization” (Table 3), with *p* values of 0.04, 0.001, and 0.04, respectively. Regarding all areas that were identified by the JABPE, the number of new private universities or schools was higher than that of old private universities or schools (Table 3).

### 3.4. The Assessment of 17 Public Universities or Schools Compared with 29 Old Private Universities or Schools

To explore the difference among schools established prior to the relaxation of the standards for the establishment of universities, the assessments were compared between public universities and old private universities, as it is generally considered that deviation score of public schools is better than private schools in Japan. For “improvements”, the main differences between 17 public and 29 old private universities or schools were “2. Organization of Curriculum”, “4. Pharmaceutical Education Curriculum” and “7. Admission Policy and System for Acceptance”, with chi-square test *p* values of 0.01, 0.06, and 0.001, respectively (Table 4). For “3. Basic Contents of Medical Professional Education”, “4. Pharmaceutical Education Curriculum”, “5. Pharmacy Clerkship”, “9. Student Services” and “13. Self-check/Self-evaluation”, the number of public universities or colleges that were identified by the JABPE (as “improvements”) was higher than the number of old private universities or colleges (Table 4).

## 4. Discussion

The Standards for the Establishment of Universities in Japan were revised, and the number of pharmacy schools and universities subsequently more than 1.5 time increased over a last decade. To ensure the quality of Japanese pharmacy education, the Japan Accreditation Board for Pharmaceutical Education (JABPE) established the Third-Party Pharmaceutical Education Evaluation in 2008, and the first cycle of assessments of all Japanese pharmacy universities and schools by JABPE was completed in March 2020. It is necessary to continue the assessment of Japanese pharmacy education or education systems based on these results.

Currently, the total population in Japan is declining, and the elderly population is increasing, the number of university entrance candidates will show a greater decline in the near future [12]. In our study, with regard to “improvements”, we found a significant difference between public and private universities or schools. The number of public universities or schools with “improvements” was less than that of private universities or schools. These results suggest that new universities or schools established since 2003 has not yet established their own quality assurance mechanism within the institutions. In fact, the results of our comparison of old private universities with new private universities or schools showed that the number of old private universities or schools assessed as “improvements” was less than the number of new private universities or schools in all assessment areas. Therefore, these issues, as “improvements”, might have been triggered by the revision of the Standards for the Establishment of Universities in Japan, in which the fundamental criteria were likely relaxed. In Australia had the similar issues in 2000s, in the pursuit of mass higher education, fears were often expressed that the quality of higher education suffers as access is increased. They revealed that, whilst widening access results in more students with lower levels of academic achievement entering higher education, this does not necessarily equate to a lowering of educational quality. Furthermore, although on average student progression rates dropped slightly, retention rates actually increased in the majority of universities, suggesting high levels of student perseverance. In addition, there were already wide variations in attrition and progression rates between universities, and the changes observed did not lead to substantial alterations. However, these results are not pharmacy education, and there are already issues with 8. Grading/Promotion/Graduation in Japan. Therefore, we must continue to the research to these influences [13].

On the other hand, some assessment areas (i.e., 3, 4, and 5), particularly “4. Pharmaceutical Education Curriculum”, showed no significant, but slight differences between public and old private universities or schools (*p* = 0.06: chi-square test). These results may be due to the fact that before the four-year pharmacy education programme was revised, private universities or schools mainly led pharmacist education, and public universities or schools mainly led basic science or scientist education. In fact, assessment areas 3, 4 and 5 are items that are required for pharmacist education, and the number of old private universities or schools identified as needing improvements was less than that of public universities or schools. In addition, “2. Organization of Curriculum” and “7. Admission Policy and System for Acceptance of the Assessment Area” showed significant differences between public and old private universities or schools (*p* < 0.01; chi-square test). Notably, 7 of the assessment areas were cleared by all public universities or schools. These results suggest that public universities or schools implement appropriate operations for areas 2 and 7, which are related to the quality of education.

Areas “3. Basic content of Medical Professional Education”, “6. Education for Nurturing Problem-Solving Ability” and “8. Grading/Promotion/Graduation” were identified as “improvements” in almost all universities or schools (94.6%, 95.9% and 94.6%, respectively). In particular, for “6. Education for Nurturing Problem-Solving Ability”, we have already reported and realized the necessity of improvement [16,17]. However, areas 3 and 8 involve severe issues to ensure the quality of pharmacy education, and these issues have been revealed more clearly by the analysis of the results of JABPE’s assessments. In particular, for “8. Grading/Promotion/Graduation”, the number of university entrance examinees will decline in the near future, but it has already been decided that new universities and colleges of pharmacy will be established after 2021. Therefore, areas “7. Admission Policy and System for Acceptance” and “8. Grading/Promotion/Graduation” would become worse. The criteria for “8. Grading/Promotion/Graduation” are unique to each university or school, and they are also assessed by JABPE every 7 years. As pharmacy universities or schools conduct self-assessments to pass the JABPE’s evaluation, not self-assessment for essential improvements, preparation and improvement for the JABPE evaluation are superficial, and do not bring any essential changes to the university. Therefore, we need to review the Japanese pharmacy education system or the assessment criteria for Japanese pharmacy education to bring about essential change.

## 5. Conclusions

Currently, although we have a national examination to obtain the pharmacist license, pharmacy education does not have an outcome standard for undergraduate education or a competency framework for new pharmacists or for the renewal of pharmacist licenses to ensure the quality of pharmacists. Each university or school decides the graduation criteria for pharmacy students based on the school’s own criteria. In this study, all universities (public and private) were more or less pointed out the “improvements” by JABPE. Therefore, to ensure the quality of pharmacy students and pharmacists, it is necessary to discuss Japan’s own standards while referring to the world’s standards [18,19]. At the same time, the JABPE will have to create new assessment standards based on the assessment results of the first cycle and continue to assess pharmacy universities or colleges.

## Figures and Tables

**Table 1:** Assessment of all 74 universities or schools by Third-Party Pharmaceutical Education Evaluation.

Assessments Areas	A11 74 Universities or Schools			
	Improvements		Recommendations	
	(Numbers of Universities or Colleges)	(%)	(Numbers of Universities or Colleges)	(%)
1. Mission and Goals	22	29.7	57	77.0
2. Organization for Curriculum	45	60.8	57	77.0
3. Basic Contents of Medical Profession Education	70	94.6	64	86.5
4. Pharmaceutical Education Curriculum	57	77.0	58	78.4
5. Pharmacy Clerkship	62	83.8	50	67.6
6. Education for Nurturing Problem-Solving Ability	71	95.9	58	78.4
7. Admission Policy and System for Acceptance	34	45.9	50	67.6
8. Grading/Promotion/Graduation	70	94.6	57	77.0
9. Student Services	16	21.6	53	71.6
10. Teacher Organization/Staff Organization	27	36.5	67	90.5
11. Institutions/Facilities	3	4.1	25	33.8
12. Collaborative Relationships with Society	0	0.0	44	59.5
13. Self-check/Self-evaluation	65	87.8	59	79.7

**Table 2:** Assessments of 17 Public and 57 Private universities or colleges.

Assessments Areas	Public Universities or Schools (*n* = 17)		Private Universities or Schools (*n* = 57)		*p*-Value
	Improvements (Numbers of Universities or Colleges)	Improvements (%)	Improvements (Numbers of Universities or Colleges)	Improvements (%)	
1. Mission and Goals	3	17.6	19	33.3	0.18
2. Organization for Curriculum	4	23.5	41	71.9	0.00 **
3. Basic Contents of Medical Profession Education	16	94.1	54	94.7	0.66
4. Pharmaceutical Education Curriculum	15	88.2	42	73.7	0.18
5. Pharmacy Clerkship	14	82.4	48	84.2	0.56
6. Education for Nurturing Problem-Solving Ability	15	88.2	56	98.2	0.13
7. Admission Policy and System for Acceptance	0	0.0	34	59.6	0.00 **
8. Grading/Promotion/Graduation	15	88.2	55	96.5	0.22
9. Student Services	4	23.5	12	21.1	0.53
10. Teacher Organization/Staff Organization	2	11.8	25	43.9	0.01 *
11. Institutions/Facilities	0	0.0	3	5.3	0.45
12. Collaborative Relationships with Society	0	0.0	0	0.0	-
13. Self-check/Self-evaluation	15	88.2	50	87.7	0.66

Note: *: *p* < 0.05; **: *p* < 0.01.

**Table 3:** Assessment of Old and New Private Universities or Schools.

Assessments Areas	Old Private Universities or Schools (*n* = 29)		New Private Universities or Schools Since 2003 (*n* = 28)		*p*-Value
	Improvements (Numbers of Universities or Colleges)	Improvements (%)	Improvements (Numbers of Universities or Colleges)	Improvements (%)	
1. Mission and Goals	8	27.6	11	39.3	0.26
2. Organization for Curriculum	19	65.5	22	78.6	0.21
3. Basic Contents of Medical Profession Education	26	89.7	28	100.0	0.13
4. Pharmaceutical Education Curriculum	18	62.1	24	85.7	0.04 *
5. Pharmacy Clerkship	23	79.3	25	89.3	0.25
6. Education for Nurturing Problem-Solving Ability	28	96.6	28	100.0	0.51
7. Admission Policy and System for Acceptance	12	41.4	22	78.6	0.00 **
8. Grading/Promotion/Graduation	27	93.1	28	100.0	0.25
9. Student Services	6	20.7	6	21.4	0.6
10. Teacher Organization/Staff Organization	9	31.0	16	57.1	0.04 *
11. Institutions/Facilities	1	3.4	2	7.1	0.49
12. Collaborative Relationships with Society	0	0.0	0	0.0	-
13. Self-check/Self-evaluation	24	82.8	26	92.9	0.23

*: *p* < 0.05; **: *p* < 0.01.

**Table 4:** Assessment of Public and Old Private Universities or Schools.

Assessments Areas	Public Universities or Schools (*n* = 17)		Old Private Universities or Schools (*n* = 29)		*p*-Value
	Improvements (Numbers of Universities or Colleges)	Improvements (Numbers of Universities or Colleges)	Improvements (Numbers of Universities or Colleges)	Improvements (%)	
1. Mission and Goals	3	17.6	8	27.6	0.35
2. Organization for Curriculum	4	23.5	19	65.5	0.01 *
3. Basic Contents of Medical Profession Education	16	94.1	26	89.7	0.53
4. Pharmaceutical Education Curriculum	15	88.2	18	62.1	0.06
5. Pharmacy Clerkship	14	82.4	23	79.3	0.56
6. Education for Nurturing Problem-Solving Ability	15	88.2	28	96.6	0.31
7. Admission Policy and System for Acceptance	0	0.0	12	41.4	0.00 **
8. Grading/Promotion/Graduation	15	88.2	27	93.1	0.47
9. Student Services	4	23.5	6	20.7	0.55
10. Teacher Organization/Staff Organization	2	11.8	9	31.0	0.13
11. Institutions/Facilities	0	0.0	1	3.4	0.63
12. Collaborative Relationships with Society	0	0.0	0	0.0	-
13. Self-check/Self-evaluation	15	88.2	24	82.8	0.48

*: *p* < 0.05; **: *p* < 0.01.

## Data Availability

Publicly available data were analyzed in this study. This data can be found here: [https://jabpe.or.jp/]. We collected our data here (JABPE home page).

## Supplementary Materials

The following are available online at https://www.mdpi.com/2226-4787/9/1/6/s1, Table S1: Assessment of 17 Public and 29 Old Private and 28 New Private Universities or Schools.
