# Supplementary material for: A Comparative Exploration of Quality Assurance Results by the Third-Party Pharmaceutical Education Evaluation in Japan
Source: Pharmacy (Basel). 2020 Dec 31;9(1):6. doi: 10.3390/pharmacy9010006 (PMC7838897; doi:10.3390/pharmacy9010006)
Supplement: Supplementary file 1 [file pharmacy-09-00006-s001.pdf]

pharmacy

MDPI

Supplementary 1 Assessment of 17 Public and 29 Old Private and 28 New Private Universities or Schools.

Assessments Areas	All public 17 universities or colleges				All pribate 57 universities or colleges				Old pribate 29 univversities or colleges				New pribate 28 univerisites or colleges (since 2003)			
	Improve ments	Improve ments %	Recom menda tions	Recom menda tions %	Improve ments	Improve ments %	Recom menda tions	Recom menda tions %	Improve ments	Improve ments %	Recom menda tions	Recom menda tions %	Improve ments	Improve ments %	Recom menda tions (n=28)	Recom menda tions %
**1. Mission & Goals**	3	17.6	11	64.7	19	33.3	46	80.7	8	27.6	21	72.4	11	39.3	25	89.3
**2. Organization for Curriculum**	4	23.5	12	70.6	41	71.9	45	78.9	19	65.5	21	72.4	22	78.6	24	85.7
**3. Basic Contents of Medical Profession Education**	16	94.1	14	82.4	54	94.7	50	87.7	26	89.7	26	89.7	28	100.0	24	85.7
**4. Pharmaceutical Education Curriculum**	15	88.2	12	70.6	42	73.7	46	80.7	18	62.1	23	79.3	24	85.7	23	82.1
**5. Pharmacy Clerkship**	14	82.4	9	52.9	48	84.2	41	71.9	23	79.3	22	75.9	25	89.3	19	67.9
**6. Education for Nurturing Problem Solving Ability**	15	88.2	13	76.5	56	98.2	45	78.9	28	96.6	23	79.3	28	100.0	22	78.6
**7. Admission Policy and System for Acceptance**	0	0.0	10	58.8	34	59.6	40	70.2	12	41.4	18	62.1	22	78.6	22	78.6
**8. Grading / Promotion / Graduation**	15	88.2	13	76.5	55	96.5	44	77.2	27	93.1	18	62.1	28	100.0	26	92.9
**9. Student Services**	4	23.5	12	70.6	12	21.1	41	71.9	6	20.7	20	69.0	6	21.4	21	75.0
**10. Teacher Organization/Staff Organization**	2	11.8	14	82.4	25	43.9	53	93.0	9	31.0	26	89.7	16	57.1	27	96.4
**11. Institutions / Facilities**	0	0.0	2	11.8	3	5.3	23	40.4	1	3.4	9	31.0	2	7.1	14	50.0
**12. Collaborative Relationships with Society**	0	0.0	4	23.5	0	0.0	40	70.2	0	0.0	17	58.6	0	0.0	23	82.1
**13. Self-check / Self-evaluation**	15	88.2	14	82.4	50	87.7	45	78.9	24	82.8	23	79.3	26	92.9	22	78.6
